# Efficacy and Safety of a Fixed-Dose Combination of Etoricoxib–Tramadol Biphasic Tablet in Moderate-to-Severe Acute Pain: A Randomized, Double-Blind, Parallel-Group, Active-Controlled Trial

**DOI:** 10.3390/jcm14124327

**Published:** 2025-06-17

**Authors:** Tania A. Sibaja, Guadalupe A. Espinoza, Yazmin I. Dávila, Erick M. Salinas, Juan J. Venegas, Dany Batista, Livan Delgado-Roche

**Affiliations:** 1 Oaxaca Site Management Organization S.C., Humboldt 302, Colonia Centro, Oaxaca 68000, Mexico; 2 Centro Oncológico Potosino, La Mora 139, Fraccionamiento del Parque, San Luis Potosí 78294, Mexico; 3 Instituto de Investigaciones Aplicadas a la Neurociencia A.C., Pasteur 205, Colonia Zona Centro, Durango 34000, Mexico; 4 Independent Researcher, Nicolás Bravo Sur 712-A, Colonia Universidad, Toluca 50130, Mexico; 5 Clínica de Ozonoterapia RGH A.C., 31 Poniente 2523, Colonia Benito Juárez, Puebla 72410, Mexico; 6 Pharmometrica S.A de C.V., Avenida de Las Granjas 972, Azcapotzalco, Ciudad de México 02230, Mexico; 7 Laboratorios Liomont, S.A. de C.V., Adolfo López Mateos 68, Colonia Cuajimalpa, Cuajimalpa de Morelos, Ciudad de México 05000, Mexico

**Keywords:** etoricoxib, tramadol, multimodal analgesia, biphasic tablet, acute pain, postoperative pain

## Abstract

**Objectives:** The aim of the present study was to evaluate the efficacy and safety of etoricoxib–tramadol 120 mg/100 mg (Eto-Tr) in acute moderate-to-severe pain. **Methods:** Eto-Tr once a day (*n* = 29) or naproxen 220 mg + tramadol 50 mg (Nap-Tr) every 12 h (*n* = 28) were administered after a third molar extraction for three days. Pain intensity difference at 4 h (PID4) was determined as the primary outcome. In addition, total pain relief (TOTPAR), trismus control, and adverse events were addressed. **Results:** The population PID4 score was 0 mm (Nap-Tr IQR 13 mm; Eto-Tr IQR 35 mm; *p* = 0.182). No differences for PID scores were observed (1 h to 72 h). TOTPAR increased gradually from 35.71% (Nap-Tr) and 39.39% (Eto-Tr) at 4 h to 67.86% (Nap-Tr) and 58.62% (Eto-Tr) at 72 h. Sustained pain relief over time and clinically meaningful trismus reduction was also observed (Nap-Tr: 4 mm [IQR 28.10] vs. Eto-Tr: 9.8 mm [IQR 12.3], *p* = 0.175). Common adverse events were notified [Nap-Tr (*n* = 5, 19%); Eto-Tr (*n* = 8, 27%)]. **Conclusions:** The once-daily administration of Eto-Tr 120 mg/100 mg showed similar efficacy and safety to conventional treatment in moderate-to-severe acute pain. The once-daily regimen together with a multimodal analgesia represents a suitable patient-centered alternative for pain management.

## 1. Introduction

Effective postoperative pain management is a cornerstone of surgical care, particularly following procedures associated with significant tissue trauma, such as third molar extraction. Despite advances in analgesic therapies, postoperative pain control remains challenging due to individual variability in pain perception, surgical complexity, surgeon technique differences, and comorbidities. Notably, over 50% of patients report moderate to severe pain in the postoperative period, being their principal concern in both inpatient and ambulatory settings. This underscores the need for optimized analgesic regimens that balance efficacy, safety, and tolerability [1,2].

The acute pain model following a surgical extraction of impacted third molars was first established in the mid-1970s [3]. Over the past five decades, this model has been extensively adopted for evaluating analgesic efficacy in both clinical research and regulatory approval processes. Postoperative acute pain after impacted third molar extraction typically peaks in intensity 6–8 h post-surgery, coinciding with the local anesthetic cessation [4]. Pain levels then remain elevated for at least 12 h before gradually subsiding over the subsequent 24–48 h [5]. This predictable time-course allows researchers to assess the analgesic effect of single-dose therapies within a 12 h window or, in multi-dose regimens, to extend observations for up to 72 h.

Monotherapy with analgesics may prove insufficient for managing acute dental pain in some cases. When monotherapy fails, multimodal analgesic strategies—combining medications with distinct mechanisms of action—are often employed. Such combinations aim to achieve comparable efficacy with lower individual drug doses or reduced dosing frequency, thereby minimizing adverse effects (AEs) while enhancing pain control [6]. For example, pairing rapid-onset non-steroidal anti-inflammatory drugs (NSAIDs) with modified-release opioids such as tramadol may address multiple pain pathways from different pharmacokinetics behavior. Critically, evidence suggests that these combinations do not compromise the safety profiles of the individual agents when administered within the therapeutic window [7,8,9]. Biphasic tablets (also known as dual-release or bimodal-release tablets) are designed to deliver drugs in two distinct phases: an immediate-release (IR) dose for rapid pain relief, followed by a sustained-release (SR) dose for a prolonged therapeutic effect. This approach offers several advantages for pain management compared to conventional formulations, including (i) rapid onset of action + prolonged effect, (ii) improved patient compliance, (iii) reduced dosing frequency and side effects, (iv) flexibility in drug combinations with different mechanisms of action, (v) reduced risk of drug abuse, and (vi) better cost-effectiveness in long-term therapies [9].

Our approach, a fixed-dose combination of etoricoxib–tramadol 120 mg/100 mg in a biphasic-release tablet, leverages modified-release tramadol to enhance tolerability relative to immediate-release formulations while providing analgesic effects with a sustained and immediate-release etoricoxib. Etoricoxib’s prolonged elimination half-life (>20 h) and potent cyclooxygenase-2 (COX-2) inhibition further support once-daily administration, which may optimize pain control, maintain tolerability, and improve patient adherence compared to shorter-acting regimens [10,11]. Therefore, the aim of the present study was to address the equivalence hypothesis in terms of efficacy to demonstrate the analgesic effect of a fixed-dose combination containing Eto-Tr 120 mg/100 mg as a biphasic tablet for once-daily administration, together with the safety and tolerability evaluation of the test intervention.

## 2. Materials and Methods

### 2.1. Study Design

This is a phase IIb, randomized, double-blind (investigator/staff and patients), active-controlled, parallel groups, multicenter trial. No patients or patients’ organizations were involved in the study design. Due to the difference between administration schedules (test vs. comparator), placebos were used. To ensure the blinding, all the investigational product and placebos were identical in appearance. After providing informed consent and meeting eligibility criteria, participants were randomly assigned (1:1 allocation) to one of two treatment arms using a web-based master randomization list (https://www.sealedenvelope.com/simple-randomiser/v1/lists/se_list_209919177068546, accessed: 26 February 2021) generated by the Sponsor of the study. The random allocation information was requested by the investigators or blind delegated personnel to unblind clinical research associates (CRAs). The protocol established stopping rules including serious adverse events, other patient safety issues as per investigator criteria, or by decision of the sponsor/health authorities.

Primary efficacy endpoint was the pain intensity difference at 4 h post-surgical (PID4). PID4 was calculated from pain intensity measures using a 100 mm visual analogue scale (VAS). Total pain relief (TOTPAR 1 h to 72 h) assessed via a five-point categorical pain relief scale (PAR) (0 = none; 1 = slight; 2 = moderate; 3 = significant; 4 = complete relief) [12], trismus control (defined as the difference of interincisal distance between baseline and study finalization), and rescue medication use. The frequency and characteristics of adverse events were monitored throughout the study. The researchers systematically monitored the safety issues pre-established in the protocol through a review of the patient’s diary, follow-up calls, or during in-person office visits. An equivalence design was selected to compare the experimental therapy against an active comparator with demonstrated efficacy and regulatory approval for acute postoperative pain. In addition, the selection of the active comparator was in alignment with ICH E10, which prioritizes ethical and methodological rigor by avoiding placebo groups when proven therapies exist. Equivalence margins were predefined as ±20 mm (measured by VAS), based on clinically meaningful differences previously reported [13,14]. The surgery procedures followed standardized anesthesia and perioperative care as per investigator discretion. Surgical complexity was graded as follows: Grade I (forceps extraction), Grade II (osteotomy), Grade III (osteotomy with coronal cut), and Grade IV (complex extraction).

### 2.2. Study Population and Ethics

Eligible outpatients were adults aged 18–40 years (male or female) scheduled for surgical extraction of impacted mandibular third molars (Pell and Gregory class II or III) [15]. Participants were also required to demonstrate the ability to comply with the study protocol and adhere to the prescribed treatment regimen. Exclusion criteria included clinically significant abnormal medical history; known hypersensitivity to any active ingredient or excipient of the study drugs; history of active peptic or gastro-duodenal ulcer or a diagnosis thereof within 30 days prior to informed consent; severe hepatic dysfunction (Child–Pugh class C); creatinine clearance < 30 mL/min; coagulation disorders; systemic lupus erythematosus; congestive heart failure (NYHA class II–IV); established coronary artery disease, peripheral artery disease, or cerebrovascular disease; revascularization procedures within the preceding 3 months; high risk for acute cardiovascular events (e.g., heavy smoking, uncontrolled hypertension, or diabetes mellitus); history of neoplastic diseases with systemic involvement or active chemotherapy; history of drug abuse, alcohol dependence, or tobacco use disorder; concomitant use of monoamine oxidase inhibitors use within 2 weeks prior to study treatment initiation; concomitant use of analgesics, neuromodulators, or long-acting NSAIDs that could not be discontinued (per physician judgment) for at least five half-lives prior to surgery; history of seizures or use of anticonvulsant medications that lower the seizure threshold; positive pregnancy test, breastfeeding status, or positive urine drug screen. A comprehensive list of inclusion/exclusion and withdrawal/elimination criteria is available at ClinicalTrials.gov (NCT05995912).

The trial protocol adhered to ICH E6(R2) (Good Clinical Practice) and E9 (Statistical Principles) guidelines. Study protocol, informed consent, and other materials for patients were previously reviewed and approved by Ethics Committees [(1) Oaxaca Site Management Organization CEI-OSMO-765/2020, CEI-OSMO-822/2020, (2) Hospital La Misión IIAEGA-LT-20.001, and (3) Hospital SMIQ LT-04-20]; meanwhile, authorization from the local health authority (Comisión Federal para la Protección contra Riesgos Sanitarios) was also obtained. No methodological amendments were implemented during the study. The investigation sites were located in San Luis Potosí (Centro Oncológico Potosino), Oaxaca (Oaxaca Site Management Organization S.C.), Puebla (Clínica de Ozonoterapia RGH A.C.), Estado de México (Independent Researcher), and Durango (Instituto de Investigaciones Aplicadas a la Neurociencia A.C.).

### 2.3. Study Intervention

Participants allocated in the active comparator arm (Nap-Tr) received naproxen 220 mg (Analgen^®^, Laboratorios Liomont S.A. de C.V., Mexico City, Mexico; immediate-release tablet) plus tramadol 50 mg (Tradol^®^, Grünenthal de México S.A. de C.V., Mexico City, Mexico; immediate-release capsule), every 12 h. The experimental arm (Eto-Tr) received a biphasic-release tablet containing etoricoxib 120 mg and tramadol 100 mg once daily. Both treatments were administered immediately post-surgery for three consecutive days. Postoperative rescue analgesia consisted of ketorolac 30 mg (Dolac^®^ 30, Siegfried Rhein S.A. de C.V., Mexico City, Mexico; sublingual tablet), permitted only if participants reported inadequate pain control (VAS ≥40 mm) ≥ 4 h after the initial study drug administration.

### 2.4. Statistical Analysis

Sample size was calculated based on the primary efficacy variable; the pain intensity difference (PID) measured by VAS (100 mm). The following assumptions were applied: therapeutic equivalence hypotheses, an equivalence margin of ±20 mm, a standard deviation (SD) of 20 mm (considered clinically relevant) [14,15,16,17], a two-sided type I error (α = 0.05), and 90% statistical power. The estimated sample size was 46 subjects (23 per treatment arm). Considering a potential dropout (20% anticipated rate), the final sample size was 58 subjects (29 per treatment arm). The sample size calculation was performed using the Free Analysis Research Tool for Sample Size Iterative Estimation (FARTSSIE, TO, Canada) version 2.4 (available at: http://individual.utoronto.ca/ddubins/. Accessed: 16 October 2019) [18].

Statistical analyses were conducted in the per-protocol (PP) and in the intention-to-treat (ITT) populations, as appropriate. No interim analyses were pre-established. Data comparisons were made using the Student’s *t*-test (independent samples) or the Mann–Whitney U-test, depending on data distribution and normality assumptions. A two-sided significance level of 0.05 was applied, and therapeutic equivalence (±20 mm) was evaluated using a 95% confidence interval (CI), with statistical significance defined as *p* ≤ 0.05. Descriptive statistics were used to summarize numerical variables (mean, median, standard deviation, range). Categorical variables were reported as frequencies and percentages. Analysis was performed using the IBM SPSS Statistics software version 29 (IBM, Armonk, NY, USA).

## 3. Results

### 3.1. Population and Surgery Characteristics

**Figure 1:**
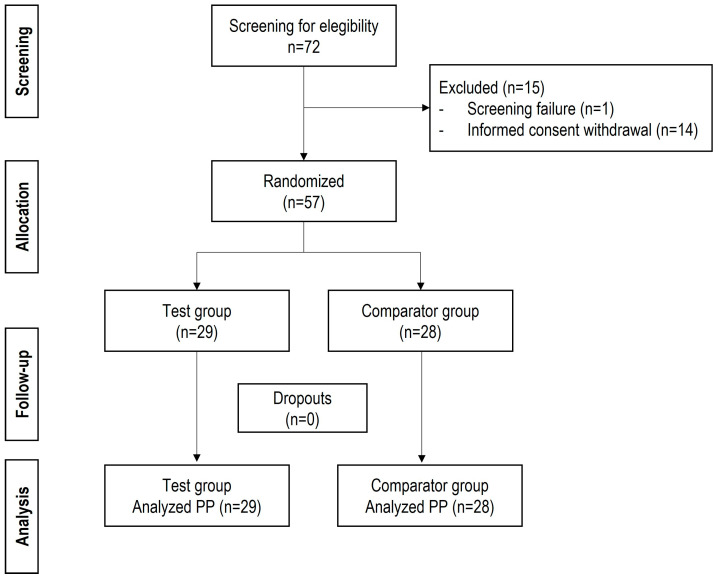
Consolidated Standards of Reporting Trials (CONSORT) flow diagram. PP: per protocol analysis.

A total of 72 patients were screened for eligibility (Figure 1). Fifteen subjects were excluded: 14 declined to participate, and one did not meet eligibility criteria. A total of 57 participants were randomized, thus meeting the calculated minimum sample size of 46 participants. Participants were allocated to either the test arm (*n* = 29) or the comparator arm (*n* = 28). Patients were recruited from five clinical sites located in Mexico. Most patients (82.75%) were treated at a site located in Oaxaca, Mexico, while the remaining participants were distributed across the other sites (Durango, San Luis Potosí, Estado de México, Puebla).

Normality assessment of baseline variables (Kolmogorov–Smirnov test, *p* > 0.05) revealed that only weight and heart rate followed a normal distribution. Nonparametric comparisons (Mann–Whitney U test, *p* > 0.05) of demographic, anthropometric, and vital sign variables revealed no significant differences between treatment groups, confirming balanced baseline characteristics—except for baseline interincisal distance (*p* = 0.023), which was significantly greater in the experimental treatment arm (Table 1). The study population had a higher proportion of women overall (57%). Surgical difficulty was predominantly grade IV (73.70% of cases), with no grade I surgeries performed. However, neither sex nor surgical difficulty grade showed a significant association with treatment allocation (Fisher’s exact test, *p* = 1.00 for sex; Chi-square test, *p* = 0.554 for difficulty grade). Median surgical times and pain intensity immediately after procedure were also equivalent between groups, which indicate homogeneity in surgical complexity, procedural duration, and basal pain across treatment arms.

### 3.2. Analgesic Efficacy

**Figure 2:**
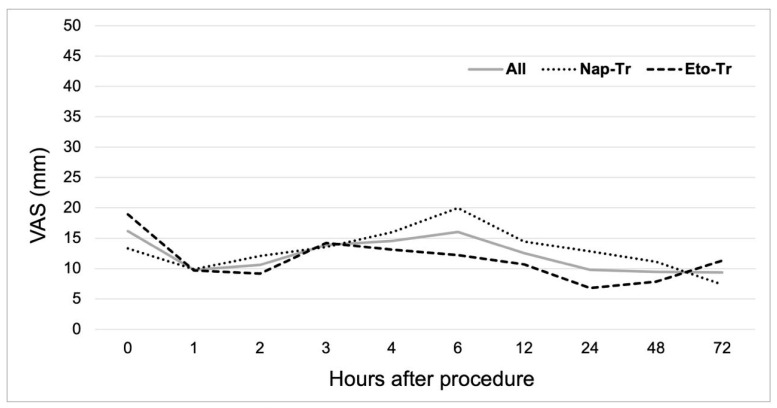
Time-course of pain intensity measured by VAS (mm) according to treatment arm and overall population. Values are the mean of pain intensity. Nap-TR, naproxen 220 mg tab + etoricoxib 50 mg cap; Eto-Tr, etoricoxib–tramadol 120 mg/100 mg biphasic tablet.

**Figure 3:**
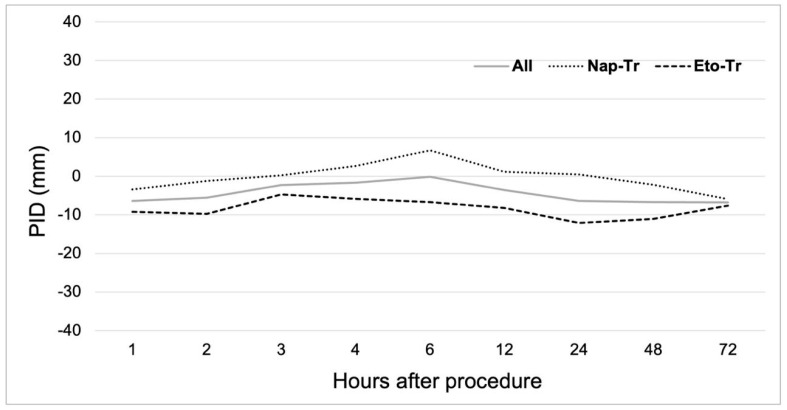
Time-course of pain intensity difference (PID) according to treatment arm and overall population. Values are the mean of PID. PID, pain intensity difference; All, overall population; Nap-Tr, naproxen 220 mg tab + tramadol 50 mg cap; Eto-Tr, etoricoxib–tramadol 120 mg/100 mg biphasic tablet.

Post-procedure pain intensity at 4 h (VAS4) for the overall population showed a median of 5 mm (IQR 24), demonstrating no significant difference from baseline pain levels recorded immediately after the procedure (*p* = 0.638). No differences between groups were observed at VAS4 or any other evaluated timepoints. To assess therapeutic equivalence between Eto-Tr and Nap-Tr, we compared the median pain intensity difference at 4 h (PID4). Both treatment arms showed median PID4 scores of 0 (Nap-Tr IQR 13; Eto-Tr IQR 35), with no statistically significant difference by Mann–Whitney U-test (*p* = 0.182). Figure 2 illustrates the VAS score time-course for the overall population and by treatment arm, while Figure 3 showed the PID time-course changes. No differences between groups were observed for PID values in any other evaluated timepoints. Of note, the use of rescue medication was not necessary during the study conduction.

**Figure 4:**
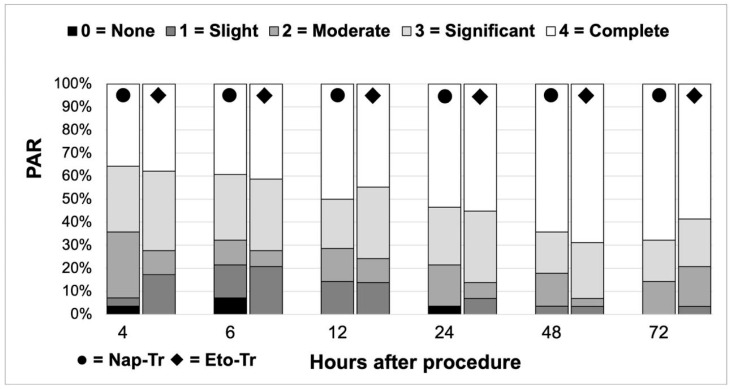
Proportional distribution of pain relief according to the PAR scale over time. PAR, pain relief; Nap-Tr, naproxen 220 mg tab + tramadol 50 mg cap; Eto-Tr, etoricoxib–tramadol 120 mg/100 mg biphasic tablet.

**Figure 5:**
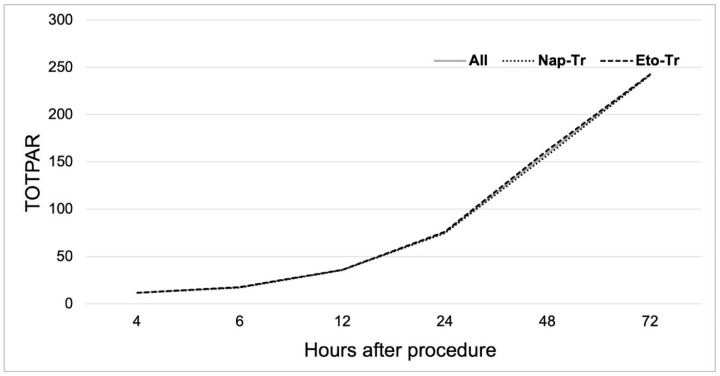
Change in TOTPAR score over time in the total population and by treatment arm. Values are the mean of TOTPAR. TOTPAR, total pain relief; Nap-Tr, naproxen 220 mg tab + tramadol 50 mg cap; Eto-Tr, etoricoxib–tramadol 120 mg/100 mg biphasic tablet.

Pain relief was evaluated through TOTPAR calculation based on the five-item PAR scale, as previously described. The mean value of PAR proportion of patients reporting complete pain relief did not differ between treatment arms at any assessed timepoint (Chi-square test, all *p* > 0.05). Complete pain relief rates increased gradually from 35.71% (Nap-Tr) and 39.39% (Eto-Tr) at 4 h to 67.86% (Nap-Tr) and 58.62% (Eto-Tr) at 72 h (Figure 4). TOTPAR analysis demonstrated sustained pain relief over time, with comparable scores between groups (Mann–Whitney U test, all *p* > 0.05). Both treatments provided clinically meaningful and sustained pain relief (Figure 5).

### 3.3. Trismus Control

Both treatments demonstrated clinically meaningful trismus reduction without significant differences between groups (Nap-Tr median: 4 mm [IQR 28.10] vs. Eto-Tr median: 9.8 mm [IQR 12.3]; Mann–Whitney U test, *p* = 0.175), demonstrating the anti-inflammatory effects. Secondary analyses showed no association between surgical duration and trismus control in the overall cohort (Spearman’s ρ, *p* = 0.084), though significant trismus variation across difficulty grades was observed (Kruskal–Wallis test, *p* < 0.001). Subgroup analysis revealed treatment-specific behavior: the Nap-Tr arm showed a procedure duration–trismus correlation (Spearman’s ρ = 0.711, *p* < 0.001) and significant trismus differences between difficulty grades (Kruskal–Wallis, *p* = 0.002). In contrast, the Eto-Tr arm maintained consistent trismus control regardless of surgical difficulty (Kruskal–Wallis, *p* = 0.750) or duration (Spearman’s ρ, *p* = 0.162).

### 3.4. Safety

Throughout the study period, all reported adverse events (AEs) were consistent with the known safety profile of the individual components comprising the fixed-dose combination. Of the 57 patients evaluated, 13 (22.8%; 11 women and two men) experienced at least one AE. A stratified analysis revealed that five subjects (19.0%) in the comparator arm reported seven AEs, while eight patients (27%) in the Eto-Tr group experienced 17 AEs. Comprehensive monitoring demonstrated no clinically relevant alterations in laboratory parameters or vital signs across either treatment group. No serious or unexpected adverse events were reported. Notably, the study achieved 100% retention, with no patient discontinuations attributable to intolerable toxicity or treatment-related tolerability concerns. These findings support that the once-daily oral fixed-dose combination of etoricoxib–tramadol 120 mg/100 mg exhibits a favorable safety and tolerability profile under the clinical study conditions.

## 4. Discussion

Postoperative pain management represents a critical challenge, particularly in procedures where moderate-to-severe pain is prevalent. Acute pain is a common clinical challenge that requires effective and safe management to improve patient outcomes and prevent complications such as chronic pain. Multimodal analgesia (MMA) has emerged as a cornerstone in acute pain management, combining different pharmacological and non-pharmacological interventions to target multiple pain pathways while minimizing side effects. MMA is based on the synergistic use of analgesics with distinct mechanisms of action including opioids and non-opioids. This approach optimizes and enhances analgesia while reducing opioid consumption, thereby lowering the risk of adverse effects (e.g., respiratory depression, nausea, and long-term dependency).

Third molar extraction, a well-established model for acute surgical pain due to its standardized inflammatory response and predictable recovery time-course, provides generalizable insights into analgesic efficacy across diverse surgical contexts. Inadequate analgesia not only heightens patient morbidity and healthcare costs but also adversely impacts quality of life [19,20]. Current guidelines endorse NSAIDs—alone or combined with acetaminophen—as a first-line therapy for moderate pain, with selective COX-2 inhibitors or weak opioids reserved for more severe cases [21,22]. However, monotherapy often proves insufficient, necessitating multimodal approaches that leverage complementary mechanisms of action to enhance efficacy while minimizing doses and AEs [22].

Our findings align with emerging evidence supporting fixed-dose combinations of COX-2 inhibitors and weak opioids for multimodal pain management. Langford et al. demonstrated that celecoxib + tramadol (44 mg/56 mg twice daily) significantly improved SPID4 in abdominal hysterectomy patients, with fewer AEs than tramadol monotherapy (14.4% vs. 23.6%) [23]. Similarly, in third molar extractions, low-dose celecoxib + tramadol (50–150 mg) showed superior efficacy to placebo and comparable AE rates to tramadol alone (12.7–15.8% vs. 29.3%) [24]. Recently, Zuqui-Ramírez et al. demonstrated the analgesic efficacy of a fixed dose combination containing immediate release etoricoxib/tramadol 90 mg/50 mg granules in patients with acute low-back pain [9]. Despite the adverse event incidence reported in the etoricoxib/tramadol group (48.1%), the treatment demonstrated early analgesic response compared with the active comparator (paracetamol/tramadol 325 mg/37.5 mg, three times a day). In the present study, a once-daily etoricoxib–tramadol 120 mg/100 mg biphasic tablet demonstrated equivalent efficacy to twice-daily naproxen 220 mg tab + tramadol 50 mg cap in terms of PID4, with mean differences within the pre-specified ±20 mm equivalence margin.

Secondary outcomes—including pain relief, trismus control, and rescue medication use—further corroborated this equivalence. Notably, the mean pain intensity and relief from the baseline was sustained during the intervention, with no significant differences in VAS, PID, or TOTPAR scores. These results suggest an adequate overlap between the inherent analgesic effect of local anesthesia during the procedure and the postoperative oral multimodal analgesia. Anesthesia plays a critical role in facilitating a positive perioperative experience and early recovery for patients. To prevent the possible analgesic carryover effect of local anesthetics and minimize their impact on the primary outcome variable, short-acting anesthetics were used (e.g., lidocaine 2% and mepivacaine 3%).

A notable distinction emerged in the relationship between surgical complexity and analgesic efficacy. While the Nap-Tr arm exhibited significant correlations between trismus control and both surgical duration and difficulty grade, the Eto-Tr arm maintained consistent efficacy irrespective of these variables. This suggests that etoricoxib’s anti-inflammatory potency may provide more robust pain modulation, independent of procedural stressors—a finding with potential implications for heterogeneous surgical populations.

In addition to the MMA, the bilayer/biphasic tablet technology of Eto-Tr is an innovative form of conventional oral drug delivery systems. Bilayer/biphasic tablets are enabling the development of different drug release profiles (immediate release + sustained release or controlled release, etc.) and to avoid potential interactions between the incompatible substance by separating them physically [25]. In a recent PK study in healthy subjects, our group demonstrated no drug interactions between etoricoxib and tramadol when a fixed drug was tested. The following PK data was obtained: Etoricoxib Cmax = 1979.29 ng/mL, Tmax = 4.20 h, AUC_0-t_ = 45714.98 h × ng/mL, T_1/2_ = 23.62 h, and Tramadol Cmax = 183.93 ng/mL, Tmax = 7.21 h, AUC_0-t_ = 2961.69 h × ng/mL, T_1/2_ = 7.77 h (unpublished data). The biphasic system is used mostly when the onset of action needs to be achieved quickly (etoricoxib) and it is followed by a prolonged release phase (tramadol), avoiding the repeated administration of drugs. This type of drug delivery is mainly suitable for analgesics, among others. While the immediate release of etoricoxib guarantees the analgesic onset, the controlled release of tramadol has the potential to provide a more constant plasma concentration and clinical effects, less frequent peak to trough fluctuations, and, possibly, fewer side effects.

Safety outcomes mirrored the known profiles of both components, with no unexpected AEs or clinically significant laboratory/vital sign changes. Retention was 100%, underscoring the tolerability of both regimens. These results position etoricoxib–tramadol as a promising alternative for acute postoperative pain, particularly in contexts requiring predictable efficacy across variable surgical complexities. Future studies should evaluate its cost-effectiveness and generalizability to other surgical models, such as orthopedic or abdominal procedures, where inflammatory cascades similarly drive postoperative pain.

The present clinical study has limitations that must be acknowledged. First, the present study did not demonstrate superiority in adherence-related metrics (no measured), its simplified dosing schedule could be clinically advantageous. Further studies with larger cohorts and longer follow-up are warranted to explore adherence benefits that may not have been assessed due to the short follow-up time. Second, procedural outcomes—such as pain time-course and trismus control—are inherently influenced by surgeon skill and technical variability. Future studies should incorporate standardized surgical protocols, including local anesthesia and perioperative care to mitigate potential biases.

In summary, MMA represents a paradigm shift in acute pain management, emphasizing efficacy and safety through combination therapies. Innovations, including biphasic tablets and other oral forms, promise to further personalize and improve pain relief while addressing treatment adherence. Continued research and interdisciplinary collaboration will be key to advancing this field.

## 5. Conclusions

The once-daily fixed-dose combination of an etoricoxib–tramadol 120 mg/100 mg biphasic tablet demonstrated efficacy and safety in patients with postoperative moderate-to-severe acute pain. Its simplified dosing regimen and consistent performance across variable surgical complexities is a practical, patient-centered alternative for acute pain management. Further studies should validate its broader applicability in other pain settings.

## Figures and Tables

**Table 1:** Baseline variables of the general population and by treatment arm.

	Total	Nap-Tr	Eto-Tr
Population distribution, *n* (%)	57 (100)	28 (49.10)	29 (50.90)
Age Years, M (IQR)	26 (6)	26.50 (6)	26 (7)
Sex Female, *n* (%)	34 (59.60)	17 (60.70)	17 (58.60)
Weight kg, $\bar{x}$ (σ)	68.97 (14.56)	67.27 (12.85)	70.60 (16.09)
Temperature °C, M (IQR)	36.20 (0.45)	36.15 (0.58)	36.20 (0.40)
Heart Rate bpm, $\bar{x}$ (σ)	74.39 (7.89)	73.61 (7.47)	75.14 (8.34)
Respiratory Rate bpm, M (IQR)	18 (4)	18 (3)	18 (4)
SBP mmHg, M (IQR)	110 (14)	110 (19)	110 (10)
DBP mmHg, M (IQR)	70 (19)	70 (13)	70 (15)
Difficulty			
I, n (%)	0 (0.00)	0 (0.00)	0 (0.00)
II, n (%)	4 (7.00)	2 (7.10)	2 (6.90)
III, n (%)	11 (19.30)	7 (25.00)	4 (13.80)
IV, n (%)	42 (73.70)	19 (67.90)	23 (79.30)
Procedure time min, M (IQR)	37(13)	38 (21)	37 (10)
Basal pain (VAS) mm, M (IQR)	5 (23)	5 (15)	10 (30)
Basal interincisal distance mm, M (IQR)	32.80 (9.10)	35.50 (14.55)	30.40 (21.90)

Nap-Tr, naproxen 200 mg tab + tramadol 50 mg cap (comparator arm); Eto-Tr, etoricoxib–tramadol 120 mg/100 mg biphasic tablet (test arm). bpm, beats per minute or breaths per minute; IQR, interquartile range; kg, kilogram; m, meter; M, median; mmHg: millimeters of mercury; VAS, visual analogue scale; $\bar{x}$, mean; σ, standard deviation; °C, Celsius degrees; SBP, Systolic Blood Pressure; DBP, Diastolic Blood Pressure.

## Data Availability

Data is unavailable due to privacy or ethical restrictions; however, the general information of the study is publicly available at ClinicalTrials.gov (NCT05995912).

## Abbreviations

The following abbreviations are used in this manuscript:

AE	Adverse event
AUC	Area under the curve plasmatic concentration vs. time
CI	Confidence interval
Cmax	Maximum plasma concentration
COX-2	Ciclooxynease-2
Eto	Etoricoxib
IQR	Interquartile range
Nap	Naproxen
NSAID	Nonsteroidal anti-inflammatory drugs
PAR	Pain relief
PID	Pain intensity difference
PP	Per protocol analysis
T1/2	Half-life
Tmax	Time to peak drug concentration
TOTPAR	Total pain relief
Tr	Tramadol
VAS	Visual analogue scale
